# The relationship between Empathy and listening styles is complex: implications for doctors in training

**DOI:** 10.1186/s12909-024-05258-9

**Published:** 2024-03-08

**Authors:** Amir Beheshti, Farzin Tahmasbi Arashlow, Ladan Fata, Farzaneh Barzkar, Hamid R. Baradaran

**Affiliations:** 1 Centre for Educational Research in Medical Sciences (CERMS), Department of Medical Education, School of Medicine, Iran University of Medical Sciences, Tehran, Iran; 2 Department of Urology, School of Medicine, Shahid Beheshti University of Medical Sciences, Tehran, Iran; 3 Medical Students Research Center (MSRC), Iran University of Medical Sciences, Tehran, Iran

**Keywords:** Empathy, Listening styles, Medical education, Communication, Medical students

## Abstract

**Background:**

Effective communication is the key to a successful relationship between doctors and their patients. Empathy facilitates effective communication, but physicians vary in their ability to empathize with patients. Listening styles are a potential source of this difference. We aimed to assess empathy and listening styles among medical students and whether students with certain listening styles are more empathetic.

**Methods:**

In this cross-sectional study, 97 medical students completed the Jefferson scale of Empathy (JSE) and the revised version of the Listening Styles Profile (LSP-R). The relationship between empathy and listening styles was assessed by comparing JSE scores across different listening styles using ANOVA in SPSS software. A p-value less than 0.05 was considered significant.

**Results:**

Overall, the students showed a mean empathy score of 103 ± 14 on JSE. Empathy scores were lower among clinical students compared to preclinical students. Most of the medical students preferred the analytical listening style. The proportion of students who preferred the relational listening style was lower among clinical students compared to preclinical students. There was no significant relationship between any of the listening styles with empathy.

**Conclusion:**

Our results do not support an association between any particular listening style with medical students’ empathic ability. We propose that students who have better empathetic skills might shift between listening styles flexibly rather than sticking to a specific listening style.

## Background

Physicians must communicate effectively with their patients to achieve the shared goal of the clinical encounter: favorable clinical outcomes [1]. Effective communication can improve patient engagement and satisfaction [1, 2]. The doctors’ communication skills and empathy are among the most critical factors for a satisfying patient-physician relationship [3].

Empathy is the ability to take the other person’s perspective during a conversation [4]. It is a multi-faceted process composed of affective and cognitive aspects [5]. While the affective dimension involves ‘feeling’ how the other person feels, the cognitive element of empathy involves understanding the person’s situation and how it has impacted their feelings [6]. More empathetic physicians are at a lower risk of burnout; their patients experience less distress and are more satisfied with care [7]. A closely related skill to empathy is active listening, defined as ‘listening and responding to another person in a way that facilitates mutual understanding’ [8].

Some studies suggest that physicians may find active listening hard [9]. Active and reflective listening requires the physician to focus on the emotional and personal aspects of the patient’s complaints [10]. It involves actively listening to patients, understanding their emotions and perspectives, and responding in a way that shows empathy and understanding [10].

People tend to have different listening preferences. Some people prefer to listen for facts or statistics, while others prefer personal examples. Some prefer to concentrate on content, while others prefer concise and ‘to the point’ presentations [11–14]. This variation reflects attitudes, beliefs, and predispositions toward the ‘how’, ‘where’, ‘when’, ‘who’, and ‘what’ of the information reception and encoding process. This concept is collectively referred to as ‘listening style’ [11, 15].

The listening styles profile is a practical approach to studying individual listening preferences [13, 16]. This approach categorizes listening preferences based on the focus of information gathering into ‘people-oriented(or relational)’, ‘action-oriented(or functional)’, ‘content-oriented(or analytical)’, and ‘time-oriented(or time)’. People tend to have a combination of preferred listening styles.

The revised version of the tool categorizes listening styles into relational, analytical, time-oriented, and critical [16]. Individuals using the relational listening style tend to care more about others’ feelings and emotions. They try to find areas of common interest with others and respond to their emotions. Functional listeners have a preference for receiving concise, error-free presentations. They are impatient and are easily frustrated when listening to a disorganized presentation. Individuals endorsing analytical listening styles prefer receiving complex and challenging information. They try to evaluate facts and details carefully before forming judgments and opinions. Time-oriented listeners reflect a preference for brief or hurried interactions with others. They tend to let others know how much time they had to listen or tell others how long they had to meet [16].

A study on the correlation between listening styles and active empathetic listening found strong connections between relational listening styles and the three stages of active empathetic listening (AELS). Analytical listening styles were found to be strongly correlated with processing and responding in the AELS, while functional listening styles were strongly correlated with processing in the AELS [12].

Research has shown that the listening styles of patients are linked to their medical communication competence, influencing their information exchange and socioemotional communication in healthcare settings [17]. Whether or not the personal preference of certain listening styles affects a physician’s ability to empathize can reveal valuable information regarding the nature of empathy and the reasons behind individual differences in empathic ability among physicians [14, 18, 19]. This information can have educational implications by shedding light on the process of empathy and how it is affected by listening styles at different stages of medical training. In other words, developing certain listening styles may be a potential educational target for teaching empathy.

We aimed to examine whether the medical students’ empathy levels and listening styles were related. We also aimed to assess the level of empathy and distribution of listening styles among medical students at different levels of their training.

## Methods

### Design, participants, and setting

All medical students(*n* = 1146) at Iran University of Medical Sciences, Tehran, Iran were eligible to participate in the study. In this cross-sectional study, 100 medical students were randomly selected and contacted and random numbers based on the students’ matriculation numbers in the university’s educational registry system. Participants were selected proportionately from different stages of their undergraduate medical training, including preclinical, clerkship, and internship. Gender was also considered when selecting the students so that the proportion of men and women would be similar at all stages.

Undergraduate medical training in Iran takes seven years and consists of four stages: basic sciences(5 semesters), pathophysiology(4 semesters), clerkship(4 semesters), and internship(3 semesters). The graduates receive an MD degree and are allowed to practice as general practitioners and family physicians. They may also get into residency programs to receive further training as specialists.

All participants completed a paper-based Persian questionnaire that consisted of the Jefferson Scale of Empathy (JSE) and the revised version of the Listening Styles Profile [20–22]. The participants also answered questions regarding sex, age, and marital status. The study protocol was approved by the institutional review board of the authors’ affiliated institution. All procedures conformed to the tenets of the Declaration of Helsinki.

### The Jefferson scale of empathy

The Jefferson scale of empathy is a 20-item questionnaire that measures empathy in the clinical setting [23]. The respondents rate each item on a 7-point Likert scale from ‘strongly disagree’ to ‘strongly agree’. The instrument has been adopted widely and has been shown to be valid and reliable in different contexts and across genders [21]. It measures empathy in 3 subscales(factors): ‘perspective taking, compassionate care, and ability to stand in patients’ shoes’. The student version of JSE was translated to Persian and validated by Shariat, et al. and showed an acceptable level of reliability [20]. This tool is the most widely adopted tool measuring empathy in the clinical setting [24]. Thus, the use of this tool would allow for an understanding of the nature of empathy as is discussed in the available literature on this subject.

### The listening styles profile

The Listening Styles Profile (LSP) is a self-administered questionnaire containing 20 questions that is designed to assess four different approaches to gathering information: people-oriented, action-oriented, content-oriented, and time-oriented styles. This questionnaire was initially designed by Watson in 1995; A revised version-which is used in the current study- was developed by Graham et al. in 2013 [10, 13, 16]. The revised version categorizes listening styles into relational, analytical, task-oriented, and critical. The tool was translated to Persian and validated in 2017 by Fatehi for use in medical sciences students and showed a Cronbach’s alpha of 0.72 [25]. Each listening style is scored based on six questions and a 7-point Likert scale yielding total scores that range from 0 to 42 [25].

### Statistical analysis

The sample size was calculated using the formula for correlational studies. Assuming a coefficient of 0.280 based on previous studies, a confidence level of 95%, and a power of 80, the number 98 was calculated [12].

Descriptive statistics were employed, including mean ± SD for continuous outcomes and rate(percent) for categorical variables, to present our data. Mean scores ± SD for JSE and mean scores ± SD for all listening styles were calculated for all participants. Means of JSE scores across students at different educational stages were compared using ANOVA. Mean JSE scores were compared between men and women using the student’s T-test. The correlation between listening styles and empathy scores was assessed using linear regression.

## Results

### Participants’ characteristics

A total of 97 medical students agreed to participate in this study. The sample comprised 51(52.6%) women and 46(47.4%) men. Participants’ age ranged from 14 to 29 years (Mean = 22, Standard Deviation(SD) = 3). Fifty-four students were living in Tehran, and 43 students were from other parts of the county living in the dormitory. The characteristics of the participants based on their educational stages are shown in Table 1.

**Table 1:** Characteristics of medical student participants

		Preclinical n(%)	Clerkship n(%)	Internship n(%)
All participants		37 [38]	31 [32]	29 [30]
Gender	Male(*n* = 46)	18(48.6)	15(48.4)	13(44.8)
	Female(*n* = 51)	19(51.4)	16(51.6)	16(55.2)
Marital Status	Single(*n* = 90)	37(100)	30(96.8)	23(79.3)
	Married(*n* = 7)	0(0)	1(3.2)	6(20.7)
Place of Residence	Home(*n* = 54)	19(51.4)	23(74.2)	12(41.4)
	Dormitory(*n* = 43)	18(48.6)	8(25.8)	17(58.6)

### Empathy

Means and standard deviations of empathy scores in each stage are reported in Table 2. The mean empathy score among participants was 103 ± 14. Although mean empathy scores were slightly higher among women compared to men, the difference in means was not statistically significant (Mean Difference = 2, 95%CI [-3.84 to 7.84], p-value = 0.08). As can be noticed in Table 2, mean empathy scores were lower in students at later stages of their training than in preclinical students. This difference was statistically significant(p-value = 0.01).

**Table 2:** Medical student participants’ empathy scores based on their sex and training stage

Gender Training Stage	Men Mean (SD)	Women Mean (SD)	Total Mean (SD)
Preclinical(*n* = 18)	108.61 (13.84)	110.68 (11.58)	109.67 (12.59)
Clerkship(*n* = 15)	98.93 (18.1)	107.18 (12.76)	103.19 (15.87)
Internship(*n* = 13)	97.84 (13.30)	95 [13]	96.27 (12.98)

### Listening styles

**Fig. 1:**
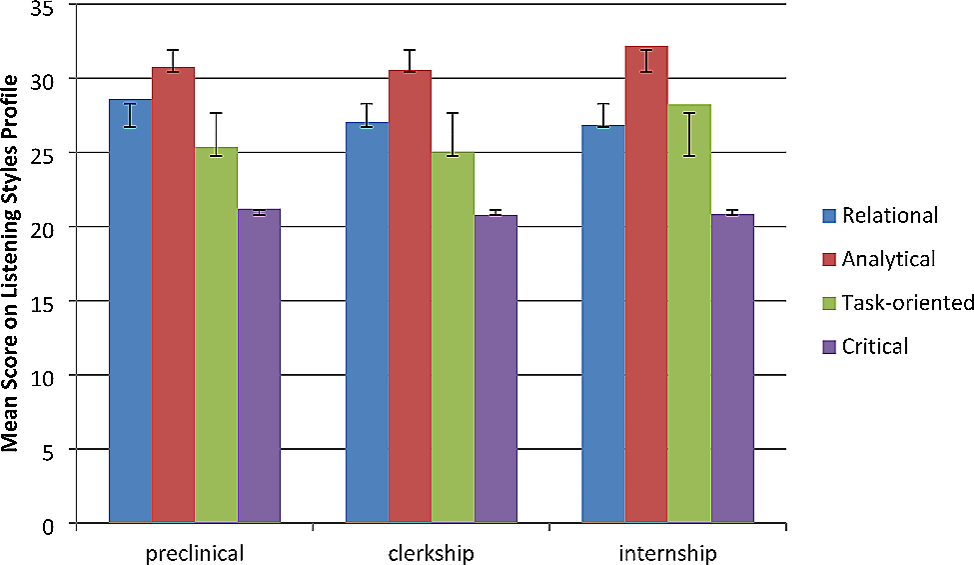
Listening styles among medical student participants at different stages of their training. The vertical axis shows the mean scores acquired by the students’ group in each listening style

Analytical listening obtained the highest mean score among medical students (Mean 31, 95% CI [29.8, 32.2]), followed by relational listening style (Mean: 27, 95% CI [26.2, 27.8]). The distribution of listening styles did not differ based on participant gender. The distribution of listening styles in participants at different stages of their training is presented in Fig. 1. Relational listening scores were lower among clinical compared to preclinical students, while analytical and task-oriented listening styles were higher in clinical students, and critical listening style remained constant at about 20 across all training stages. None of the observed trends in listening styles across training stages were statistically significant.

Empathy scores were not significantly correlated with any type of listening style. The correlation coefficients for empathy scores with each of the listening styles are presented in Table 3.

**Table 3:** Correlation between empathy and listening styles

		Relational	Analytical	Task-oriented	Critical
Empathy Score	Pearson correlation	0.131	-0.095	-0.101	0.006
	P-value	0.20	0.35	0.32	0.95

## Discussion

Both empathy and effective listening play an important role in the physician-patient relationship [5, 26, 27]. Contrary to expectations, that higher levels of empathy would be associated with a preference for a relational or people-oriented listening style, our results showed that no particular listening style was associated with higher empathy scores among medical students. This finding may be routed in differences in nature between empathy and listening styles. While relational listening style reflects the degree of concern for the patients’ emotions, the JSE measures empathy as a stable cognitive ability that is modified by the physician’s skills in perspective taking, compassionate care, and putting oneself in patient’s shoes in contrast to empathic concern which is the affective component of empathy [5, 12].

One study that assessed listening styles and empathic listening among nursing students reported that a preference for people-oriented listening style was associated with all three components of empathic listening: sensing, processing, and responding; Content-oriented listening style was correlated with processing and responding components of empathic listening. And, action-oriented listening style was strongly correlated with the processing element of empathic listening. Thus, it can be inferred that empathic listening encompasses a combination of listening styles that are used flexibly rather than a fixed preference for the relational or people-oriented listening styles [12, 17]. Moreover, listening styles are more state-related and contextual constructs in contrast to empathy that is more stable; In other words, people tend to have multiple preferred listening styles they employ in different settings [11].

Neuroscience research also provides evidence that empathy is a complex phenomenon that involves multiple components executed by different parts of the brain with distinct functions. The right temporal lobe, where mirror neurons reside, has been shown to be activated during the process of perspective empathy [28]. The posterior part of the inferior frontal gyrus is activated when we are trying to understand the intentions of others [29]. Anterior part of insula and anterior cingulate gyrus are activated when perceiving empathic distress [30]. The fact that distinct brain areas are involved in different components of empathy, is also in alignment with the idea that empathic skills constitute a complex set of cognitive abilities including different areas related to listening rather than a simple concern for others mediated by mirror neurons as in the relational listening style.

Medical students who participated in our study preferred analytical listening styles; Although they scored above 20 in all listening styles, showing a moderate tendency to use each of them. Research suggests that most individuals have a combination of preferred listening styles which may change over time [10, 11, 16]. The choice of specific listening styles is based on personal habit and preferences modulated by factors such as sex and gender roles as well as demands from the working environment [11]. While the students in our study scored lower on empathy and relational listening style at later stages of their education, interns scored higher on analytical listening style compared to preclinical students. The participants in our study scored high on analytical listening style which according to previous research contributes to careful assessment of different aspects of an issue and perspective taking [16].

According to our findings, although women scored higher in JSE-S than men, the difference was not statistically significant. Patterns of listening styles were also different between men and women. Although small in size, sex-related differences in empathy and patterns of listening styles have been well recognized and studied [11]. Men tend to prefer content and action-oriented listening styles while women show a stronger preference for people- and action-oriented styles [31, 32]. These differences have been attributed to both gender-roles and biological differences [25, 32]. In other words, independent of biological sex, people with communal gender roles prefer relational and people-oriented listening in contrast to people with agentic gender roles who prefer action-, content-, and time-oriented listening styles [11].

Considering different educational stages, we observed that students at later stages of their training scored significantly higher in task-oriented listening style. Mean empathy score was also significantly lower among participants in clinical training compared to preclinical students. Several studies have shown a decline in students’ empathy during medical school training. The literature suggests some reasons for this trend: (1) empathetic disengagement due to heavy emotional load associated with clinical encounters during training [33], (2) lack of emotional literacy, (3) gradual sensory desensitization to patients’ pain as a result of continuous exposure [33], and (4) empathy is a protective factor for burnout. Other reasons include mental health challenges such as depression and burnout that arise during medical training and have been shown to affect empathic capacity, a problem-solving in contrast to relational culture in medical schools, and higher workloads which are a barrier to communication with patients [34–37].

Several ways have been suggested to improve listening skills in health care providers can. One study emphasizes inclusion of a listening skills course in the medical school curriculum that provides advanced communication training [38]. They emphasized teaching of active and empathetic listening, as well as the use of nonverbal cues and reflective techniques. Minimizing distractions, validating patients’ feelings, and promoting cultural competence are also noted as essential components of effective listening. The article also suggests receiving feedback and engaging in self-reflection for life-long learning of listening skills [38]. To further improve listening skills among medical students, it is essential to provide explicit training in clinical reasoning and communication skills, as well as to incorporate interactive methods of teaching, such as simulated patients and case-based role-plays [39, 40]. The integration of clinical reasoning with communication skills training has also been proposed as a solution to students’ confusion over their choice between attentive listening for emotions and listening for problem-solving [41].

The participants in our study scored an overall mean of (M = 103 ± 14) on the JSE. This is very close to the findings of previous studies from Iran and other Asian countries while lower than scores of medical students in the US, Mexico, and Portugal. This difference may be attributed to cultural differences in healthcare systems, the medical school admission criteria, and educational programs across countries. Another explanation that physicians who are affected by burnout score lower on the JSE; Thus, the lower empathy scores in our study are related to higher rates of burnout in Middle-Eastern and Asian countries [35, 42, 43].

In concluding this study, one should consider some limitations. First, given that our sample consisted of ninety-seven medical students attending the same university, the study would have benefited from a greater number of participants at various universities in Iran. Secondly, our findings may be altered by potential confounding factors such as burnout and personal characteristics that affect empathy and we did not adjust for in our analyses [43]. Thirdly, this study was the first study that used the revised version of Graham Listening Styles Questionnaire for this purpose. Thus, findings cannot be reliably compared to other studies measuring listening style profiles using the initial version of the questionnaire. Fourthly, since empathy was assessed using JSE which measures clinician empathy in three cognitive dimensions of perspective taking, compassionate care, and walking in the patient’s shoes, our findings cannot be generalized to concepts related to the affective component of empathy including empathic concern. Moreover, we only used mean scores of empathy in order to assess the relationship between empathy and listening styles, and we did not collect detailed data on different subscales of empathy.

We suggest that future studies focus on the use of different listening styles in different settings and how the flexible use of listening styles relates to empathic skills. Specifically, studies may test whether a flexible use of listening styles is correlated with better empathy. Another suggestion would be that future studies include more students which could enable comparing different components of empathy across different listening styles. Also, future studies may want to assess the same issue from the patients’ perspective. We also suggest measuring burnout as a confounding factor in future studies. Another interesting research would involve creating and testing the efficacy of empathy training modules that focus on the flexible use of listening styles.

## Conclusions

Empathy strongly affects the relationship between doctors and patients. In the current study, empathy did not correlate with any of the four listening styles among medical students and interns. It is suggested that physicians with good communication skills, flexibly modify their listening style based on individual clinical contexts rather than preferring certain listening styles over others for all clinical situations.

## Data Availability

The datasets used and analysed during the current study are available from the corresponding author on reasonable request.
